# Supplementary material for: Inhibition of Bacterial Adhesion and Biofilm Formation by Seed-Derived Ethanol Extracts from Persea americana Mill
Source: Molecules. 2022 Aug 6;27(15):5009. doi: 10.3390/molecules27155009 (PMC9370132; doi:10.3390/molecules27155009)
Supplement: Supplementary file 1 [file molecules-27-05009-s001.zip › molecules-1842022-supplementary.pdf]

Supplementary Material

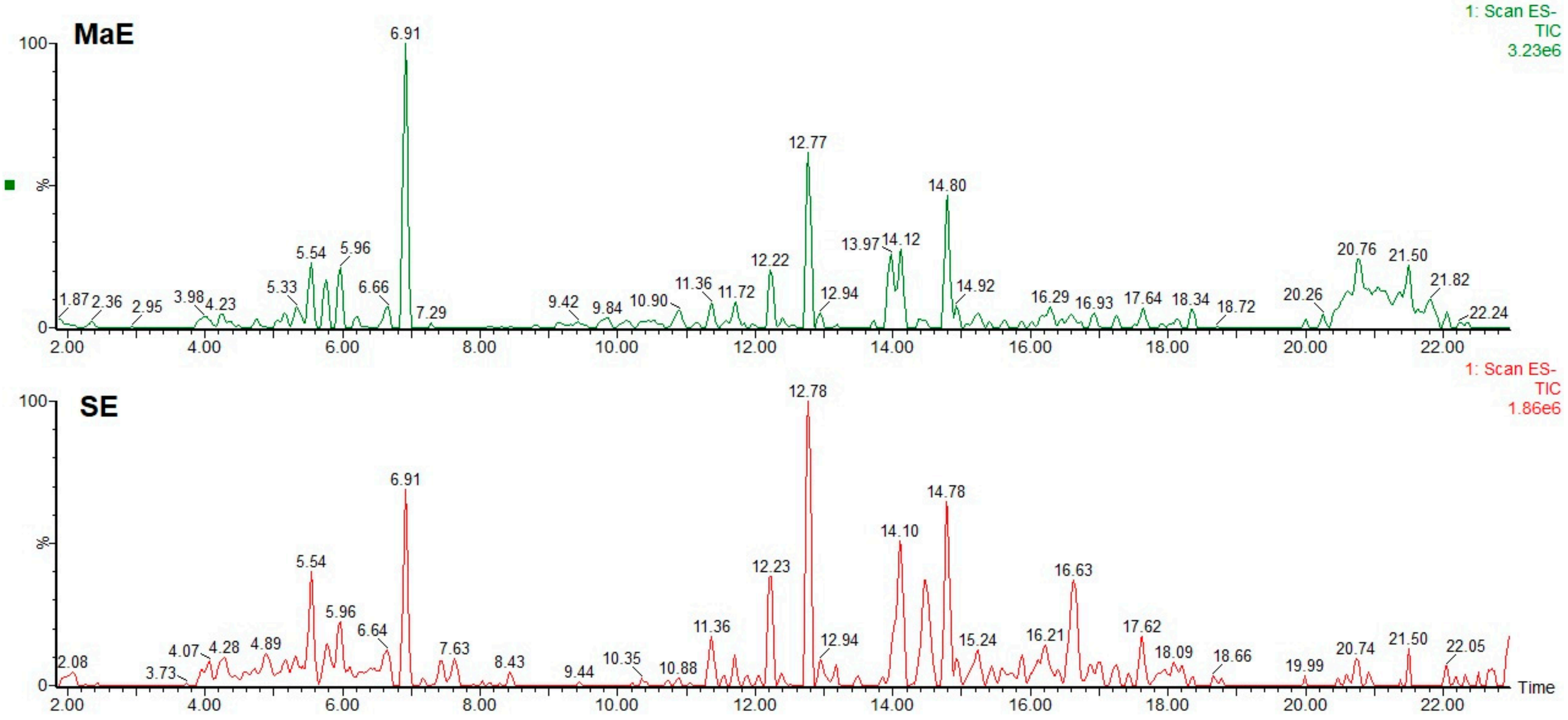

Figure S1. Chromatograms obtained from the UHPLC-DAD-QTOF analysis performed to ethanol extracts from *P. Americana* seeds.

Table S1: Retention time, assigned compounds and molecular ions obtained in the UHPLC-DAD-QTOF analysis of SE and MaE *P. americana* seed extracts, and comparison with the previous study.

Molina et al. 2022. [19]							Peaks from Figure 1S					
**Peak**	**Rt (min)**	**Mass [M-H]$^-$ (*m/z*)**	**Molecular Formula**	**Error (mDa)**	**Tentative identification**	**EPP**	**Rt (min)**	**Mass [M-H]$^-$ (*m/z*)**	**Molecular Formula**	**Error (mDa)**	**Tentative identification**	**EPP**
1	-	-	-	-	-	-	5.54	461.1620	$C_{27}H_{25}O_7$	2.0	-	***SE***, ***MaE***
2	6.90	441.1242	$C_{22}H_{18}O_{10}$	-3.4	catechin/epicatechin gallate	***SE***, ***MaE***	6.91	441.1317	$C_{22}H_{18}O_{10}$	-2.9	catechin/epicatechin gallate	***SE***, ***MaE***
3	12.20	327.1214	$C_{18}H_{16}O_6$	-2.7	Perseal C	***SE***	12.22	327.1229	$C_{18}H_{16}O_6$	-1.7	Perseal C	***SE***, ***MaE***
4	12.76	329.0969	$C_{18}H_{18}O_6$	-5.6	3',4'-Methylenedioxy-5,7-dimethylcatechin/epicatechin	***SE***, ***MaE***	12.77	329.1109	$C_{18}H_{18}O_6$	-4.1	3',4'-Methylenedioxy-5,7-dimethylcatechin/epicatechin	***SE***, ***MaE***
5	13.89	265.1602	$C_{19}H_{22}O$	0.7	-	***MaE***	-	-	-	-	-	-
6	14.10	329.2596	$C_{19}H_{38}O_4$	9.9	4,19-dihydroxynonadecanoic acid	***SE***	14.11	329.2269	$C_{19}H_{38}O_4$	5.9	4,19-dihydroxynonadecanoic acid	***SE***, ***MaE***
7	14.44	371.1578	$C_{21}H_{24}O_6$	0.2	-	***SE***	14.48	371.1858	$C_{20}H_{19}O_7$	4.5	lignan	***SE***
8	14.78	331.1248	$C_{18}H_{20}O_6$	-0.1	Perseal A	***SE***, ***MaE***	14.79	331.1637	$C_{18}H_{20}O_6$	-4.6	Perseal A	***SE***, ***MaE***
9	15.81	325.0904	$C_{19}H_{14}O_6$	1.6	p-coumaric acid glucoside	***MaE***	-	-	-	-	-	-
10	16.63	373.3218	$C_{22}H_{46}O_4$	1.7	2-hydroxy-4-oxoicosa-dienyl acetate	***SE***	16.63	373.2990	$C_{22}H_{46}O_4$	-0.3	2-hydroxy-4-oxoicosa-dienyl acetate	***SE***
11	-	-	-	-	-	-	17.62	359.0556	$C_{21}H_{11}O_6$	-3.4	-	***SE***, ***MaE***
12	20.73	345.2355	$C_{18}H_{34}O_6$	-2.1	Dideoxy-hexopyranosyl-dodecanoic acid	***SE***, ***MaE***	20.76	345.2351	$C_{18}H_{34}O_6$	-2.4	Dideoxy-hexopyranosyl-dodecanoic acid	***SE***, ***MaE***
13	21.50	383.1906	$C_{20}H_{32}O_7$	3.5	diterpene	***SE***, ***MaE***	21.50	383.1917	$C_{20}H_{32}O_7$	3.5	diterpene	***SE***, ***MaE***
